# Assessing the Biodegradation of BTEX and Stress Response in a Bio-Permeable Reactive Barrier Using Compound-Specific Isotope Analysis

**DOI:** 10.3390/ijerph19148800

**Published:** 2022-07-20

**Authors:** Tianyu Chen, Yan Wu, Jinnan Wang, Corvini François-Xavier Philippe

**Affiliations:** 1 Stake Key Laboratory of Hydrology-Water Resources and Hydraulic Engineering, Nanjing Hydraulic Research Institute, Nanjing 210029, China; chentianyu8968@outlook.com; 2 State Key Laboratory of Pollution Control and Resource Reuse, School of the Environment, Nanjing University, Nanjing 210023, China; 15261871532@163.com (Y.W.); philippe.corvini@fhnw.ch (C.F.-X.P.); 3 School of Life Sciences, University of Applied Sciences and Arts Northwestern Switzerland, 4132 Basel, Switzerland

**Keywords:** bio-permeable reactive barrier, biodegradation, BTEX, compound-specific isotope analysis, high-throughput sequencing analysis

## Abstract

By using compound-specific isotope analysis (CSIA) in combination with high-throughput sequencing analysis (HTS), we successfully evaluated the benzene and toluene biodegradation in a bio-permeable reactive barrier (bio-PRB) and the stress response of the microbial community. Under stress conditions, a greater decline in the biodegradation rate of BTEX was observed compared with the apparent removal rate. Both an increase in the influent concentration and the addition of trichloroethylene (TCE) inhibited benzene biodegradation, while toluene biodegradation was inhibited by TCE. Regarding the stress response, the relative abundance of the dominant bacterial community responsible for the biodegradation of BTEX increased with the influent concentration. However, the dominant bacterial community did not change, and its relative abundance was restored after the influent concentration decreased. On the contrary, the addition of TCE significantly changed the bacterial community, with *Aminicenantes* becoming the dominant phyla for co-metabolizing TCE and BTEX. Thus, TCE had a more significant influence on the bio-PRB than an increasing influent concentration, although these two stress conditions showed a similar degree of influence on the apparent removal rate of benzene and toluene. The present work not only provides a new method for accurately evaluating the biodegradation performance and microbial community in a bio-PRB, but also expands the application of compound-specific isotope analysis in the biological treatment of wastewater.

## 1. Introduction

Due to their neurotoxic, carcinogenic, and teratogenic properties, aromatic hydrocarbon compounds including benzene, toluene, ethylbenzene, and xylene (BTEX) isomers pose a great risk to the environment and human health. As typical BTEX compounds, benzene and toluene have frequently been detected in groundwater because of the inefficient treatment of industrial waste and its leakage [1,2,3,4].

**Figure 1:**
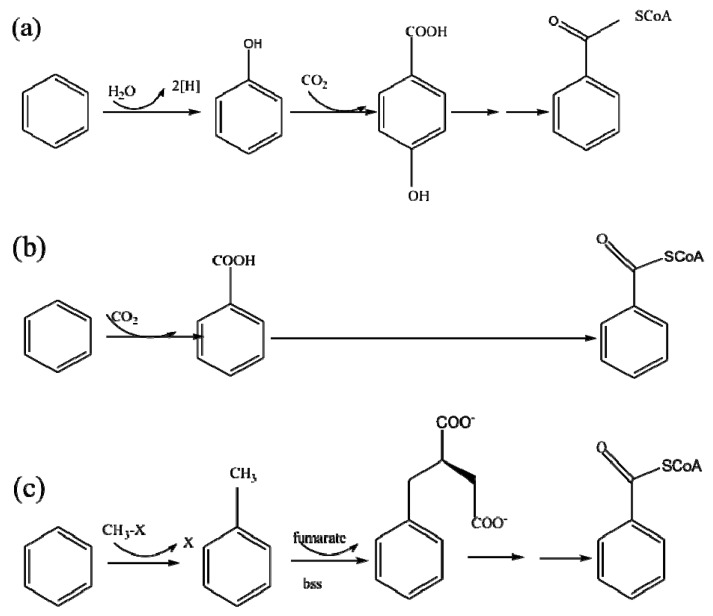
Three anaerobic degradation pathways proposed for benzene. Square brackets indicate a postulated intermediate; broken arrows indicate multiple enzymatic steps; open arrows indicate further metabolism. (**a**) Hydroxylation to form phenol, cyclohexanone, or p-hydroxybenzoate and benzoyl-CoA. (**b**) Carboxylation to form benzoate (possibly through more than one enzymatic step) and benzoyl-CoA. The carboxyl donor is unlikely to be bicarbonate but may be derived from benzene. (**c**) Alkylation to form toluene, followed by fumarate addition to form benzylsuccinate and benzoyl-CoA [41,42].

**Figure 2:**
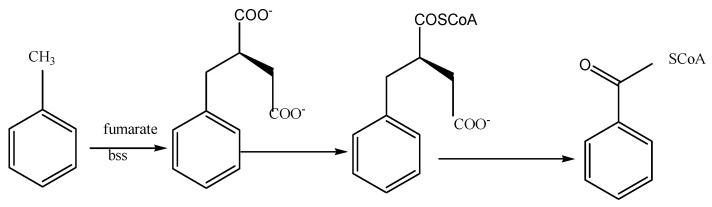
Anaerobic microbial degradation pathway of toluene. The first step in the catabolism of toluene is the addition of toluene to the double bond of fumarate to form benzylsuccinate by benzylsuccinate synthase (bss). Benzylsuccinate is then activated to CoA-thioester by a succinyl-CoA-dependent CoA-transferase, and benzylsuccinyl-CoA is then converted to succinyl-CoA and benzoyl-CoA. Benzoyl-CoA reductase initiates the degradation of benzoyl-CoA, which is thereafter further oxidized via reductive ring cleavage to carbon dioxide [43,44].

Various technologies have been developed to remove BTEX from groundwater, including physical methods (stripping, in-situ air sparging, adsorption, and filtration); chemical methods (chemical oxidation and photocatalysis); and biological methods (bioremediation, bioaugmentation in reactors, phytoremediation, and wetland restoration) [4,5,6,7,8,9]. Compared with the physical and chemical methods, which face the problem of a high operation cost and secondary pollution, bioremediation is considered a more economic and efficient method for BTEX degradation [10,11,12]. As the typical bioremediation method, the application of bio-permeable reactive barrier (bio-PRB) technology for the in situ remediation of BTEX-contaminated groundwater has attracted great interest [13]. Previous research has demonstrated that indigenous microorganisms detected in polluted groundwater/soil, such as *Actinobacteria*, *Rhodococcus*, *Arthrobacter* strains, and *Proteobacteria*, possess higher BTEX degradation activity [14,15,16,17,18,19,20,21,22,23,24]. Thus, BTEX could be adsorbed on the filler and subsequently degraded through anaerobic degradation pathways (Figure 1 and Figure 2). Many studies have determined the BTEX removal rate in bio-PRBs under different conditions in order to optimize the operation parameters [25,26,27,28,29]. However, most of these studies neglected the fact that the BTEX removal rate is not the same as the actual biodegradation rate. The BTEX removed by physicochemical transformation (e.g., dilution, dispersion, volatilization, and sorption) can be discharged into the groundwater again after a certain period of time, causing secondary pollution. Thus, optimizing the operating parameters of the biodegradation process based on the BTEX removal rate is inaccurate. On the other hand, although conventional molecular methods such as terminal restriction fragment length polymorphism, polymerase chain reaction/denaturing gradient gel electrophoresis, and cloning have been applied to determine the microbial community responsible for BTEX removal [30,31,32], the conclusions drawn from the evaluation of the BTEX removal rate in these studies were also inaccurate. In recent years, the aforementioned traditional molecular approaches have not been able to provide comprehensive insights into the structural and functional changes in the microbial community [33,34]. Thus, it is necessary to illustrate the relationship between the microbial community and the BTEX biodegradation performance.

In recent years, compound-specific isotope analysis (CSIA) has been applied to evaluate organic biodegradation through physicochemical processes according to stable isotope fractionation [35,36,37]. In addition, with the development of molecular biology technology, researchers have established high-throughput sequencing (HTS) methods, including amplicon and shotgun sequencing, with a sequencing depth and accuracy sufficient to cover complex bacterial communities [38,39]. Furthermore, since microbial activity is sensitive to environmental conditions, much attention has been paid to variations in BTEX biodegradation activity under stress conditions, especially when inhibited by high-concentration or combined pollutants [26,28,40]. As a typical volatile chlorinated hydrocarbon, TCE often co-exists with petroleum hydrocarbons [26], which could alter enzyme conformation and block essential functional groups, causing a decrease in the biodegradation capacity [40]. Thus, further investigation of the benzene and toluene biodegradation performance of bio-PRBs under stress conditions (i.e., with an increase in the influent concentration and the addition of TCE) is significant both theoretically and for engineering applications.

Herein, we report the application of CSIA in combination with HTS for the first time, a technique that can determine the relationships between the bacterial community and the actual BTEX biodegradation rate under stress conditions. This study not only provided a method for evaluating the biodegradation performance of non-degradable processes, but also helped us more accurately regulate the operational conditions of the bio-PRB to improve its biodegradation capacity.

## 2. Materials and Methods

### 2.1. Chemicals

Benzene, toluene, and TCE (trichloroethylene) were supplied by Sigma-Aldrich (China, ≥99% GR). NaOH, Na_2_SO_4_, KH_2_PO_4_, K_2_HPO_4_, CaCl_2_, and MgSO_4_ were purchased from Aladdin (China, ≥99%, AR).

### 2.2. Column Experimental Setup

**Figure 3:**
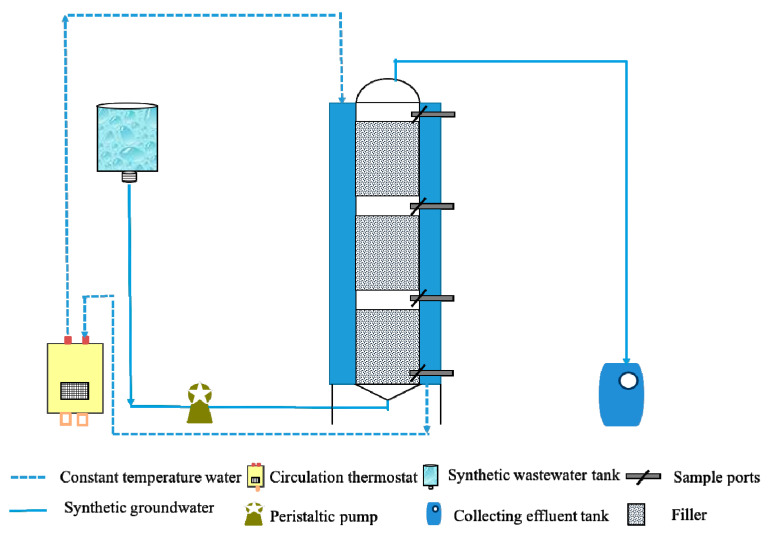
Scheme of bio-PRB instrument used in the experiments.

The column of the bio-PRB system had a working volume of 3.2 L, inner diameter of 8.0 cm, and height of 92.0 cm (Figure 3). The reactor was inoculated with sludge (50.0 g VSS L^−1^) from a secondary sedimentation tank (BASF-YPC, Nanjing, China). The composition of the simulated groundwater used in this experiment is summarized in Table S1. The start-up process lasted for 30 d until the bio-PRB system reached a stable state (Table S2), during which the influent glucose concentration was 300 mg L^−1^, with a flow velocity of 1.1 mL min^−1^. 

After the start-up process, the bio-PRB system continuously ran for a total of 200 d, which was divided into five periods. In each period, a different concentration of target pollutants (benzene and toluene) and co-existent pollutant (TCE) were added to the simulated groundwater (Table S2). The effluent of the bio-PRB system was sampled every 48 h, and the samples were collected in 40 mL standard VOC glass vials without headspace. In order to preserve isotope-containing samples, the pH was adjusted to 10 ± 0.1 with NaOH to prevent inactivation by active microorganisms. The samples were sealed and immediately stored at 4 °C before being analyzed within 7 d.

### 2.3. Batch Experiments

Batch experiments were carried out to study the biodegradation kinetics and calculate the carbon isotope enrichment factors for benzene and toluene. The actual biodegradation rates of benzene and toluene in each period could be calculated according to the carbon isotope values. The batch experiment process was conducted as follows: 50 mL sludge sample was removed from the bio-PRB at the end of each period. Then, the sludge aliquot and 430 mL of synthetic groundwater were added into a 480 mL amber boston round bottle with PTFE liner (Table S3), followed by shaking (120 rpm) at 30 °C for 48 h. Controls were first autoclaved, cooled, and spiked with benzene and toluene for corresponding treatments. The concentrations of benzene and toluene in the control sample were 10 mg L^−1^. Samples (15 mL) were taken at various time intervals in standard VOC glass vials (25 mL) for the analysis of carbon isotope values and concentration of benzene and toluene. 

### 2.4. Quantification of Benzene, Toluene, and TCE

Analyses of benzene, toluene, and TCE were conducted in accordance with US EPA Method 8260, measured with a purge and trap concentrator (Tekmar, Waltham, MA, USA) followed by gas chromatography equipped with a silica HP-5 capillary column (30 m × 0.25 mm × 0.25 μm, J&W Scientific, Folsom, CA, USA) and a flame ionization detector (GC-FID, Agilent-7820, Palo Alto, Santa Clara, CA, USA). A sample (5 mL) was introduced into the purging vessel with a syringe [45]. The sample was purged with helium gas at 35 mL min^−1^ for 11 min. The purged volatile compounds were trapped on a Tenax trap. The trap was heated to 225 °C and kept at the same temperature for 2 min to desorb benzene and toluene, which were then applied to the GC column. The operating parameters and flow rates were as follows: the injector and detector temperatures were set at 280 °C and 220 °C, respectively; the split ratio was 5:1; and the oven temperature was held at 30 °C for 3 min, then programmed at 15 °C min^−1^ to 180 °C.

### 2.5. CSIA Analysis

Compound-specific isotope analysis (CSIA) of benzene and toluene was conducted using a continuous-flow GC-C-IRMS consisting of a TRACE GC Ultra, oxidation reactor, and MAT 253 isotope ration mass spectrometer (Thermo Fisher Scientific, Waltham, MA, USA). The GC was fitted with a DB-5 capillary column (30 m × 0.25 mm × 0.25μm, J&W Scientific, Folsom, CA, USA). Benzene and toluene were extracted from samples using a Solid Period Micro Extraction (SPME) fiber at 25 °C. Benzene and toluene working standards calibrated against international reference materials for carbon isotopes were prepared and analyzed using the same method as for the real samples to correct for any isotope fractionation occurring during SPME extraction. Benzene and toluene were desorbed from the SPME fiber in the GC inlet at 270 °C then separated in the GC column. The GC temperature was held at 40 °C for 3 min then increased at 15 °C min^−1^ to 180 °C. The helium carrier gas flow through the column was 1.5 mL min^−1^. The oxidation reactor for ^13^C/^12^C was set at 940 °C. The pulse of the reference gas (CO_2_, δ^13^C: −26.42‰ VPDB) was used for the computation of the isotopic values of sample compounds. Benzene standards were analyzed three times under the same operating conditions, and the standard deviation of the measurements was typically within ±0.5‰ [46,47].

### 2.6. DNA Extraction and PCR Amplification

After the start-up process, the biofilm samples were collected via sampling ports of the bio-PRB system at the end of each operational period. Briefly, total DNA from five different biofilm samples was extracted using a FastDNA^®^ SPIN Kit for Soil (MP Biomedicals, Santa Ana, CA, USA) following the manufacturer’s instructions. DNA concentration and purity were measured by means of micro-spectrophotometry (NanoDrop^®^ ND-1000, NanoDrop Technologies, Willmington, DE, USA). 

Amplification of the 16S rRNA gene was performed with primers 27F (5′-AGAGTTTGATYMTGGCTCAG-3′) and 338R (5′-TGCTGCCTCCCGTAGGAGT-3′) for bacteria. Polymerase chain reaction (PCR) amplification was carried out in a 30 μL reaction volume containing 2 μL template DNA; 100 mM dNTP; 1 PCR buffer; 1 U of EXTaq polymerase (TransGen Biotech, Beijing, China); and 2 mM of each primer set (Table S4). Thermal cycling consisted of initial denaturation for 5 min at 94 °C; 40 cycles of denaturation at 95 °C for 30 s, annealing at 55 °C for 45 s, and elongation at 72 °C for 50 s; followed by a 5 min extension at 72 °C. 

### 2.7. Quantitative Real-Time PCR (q-PCR)

Real-time PCR was performed for all samples using oligonucleotides that were designed to target *dsrA* and *bssA*. The PCR primer sets *dsrA*f-*dsrA*r and *bssA*f-*bssA*r were specific for genes *dsrA* and *bssA* [48,49,50]. The key functional genes for dsrA and bssA were amplified using the primers DSR1F (5′-ACSCACTGGAAGCACG-3′)/DSR5R (5′-TGCCGAGGAGAACGATGTC-3′) and BSSAF (5′-ACGACGGYGGCATTTCTC-3′)/BSSAR (5′-GCATGATSGGYACCGACA-3′). The q-PCR mixture (20 mL) contained 10 mL of SYBR Premix EXTaq Super Mix (TaKaRa Japan), 0.3 mL of each primer set (10 mM), 8 mL of template DNA (5 ng/mL), and 1.4 mL of distilled H_2_O (ddH_2_O). The real-time PCR program was performed as follows: the reactions were run for 50 cycles of initial denaturation for 10 min at 95 °C, denaturation at 95 °C for 15 s, annealing for 1 min at 55 °C, and elongation at 72 °C for 20 s. In the study conducted by Rhee, DNA cloning was used to construct recombinant plasmids carrying *dsrA* and *bssA*, and five- to seven-point calibration curves (C_t_ values versus log of initial target gene copy) were generated for the q-PCRs using a 10-fold serial dilution of the plasmid [51]. In order to take into account the variation in the DNA extraction efficiency for each sample, the relative abundance of each target gene was normalized to the eubacterial 16S rRNA gene [52]. The reaction efficiency of dsrA was 85.2%, and that of bssA was 104.7%, with R^2^ values higher than 0.995 for all calibration curves.

### 2.8. Illumina High-Throughput Sequencing

The biofilm samples were sent to Majorbio Bio-pharm Biotechnoly Co., Ltd. (Shanghai, China) for Illumina high-throughput sequencing on the MiSeq platform (Illumina, San Diego, CA, USA). Raw sequences were generated using the sequencing strategy of Index 101 PE (paired-end sequencing, 101-bp reads and 8-bp index sequence). The quality control (QC) pipeline was applied to remove the adaptor at the end of reads. Unknown nucleotides of the raw sequences were removed first, and then Galaxy (http://usegalaxy.org/, accessed on 1 October 2021) was used to conduct a stricter filtration. In order to ensure that each filtered read had a high Illumina quality score, quality formats were converted and low-quality sequences were removed.

The filtered Illumina reads of the five biofilm samples were processed and analyzed on http://www.i-sanger.com/ (accessed on 1 October 2021). The results were subsampled into 35, 128 sequences (i.e., the number of sequences in the sample with the least number of sequences). The confidence threshold of 97% recommended by the RDP was applied to strictly assign the sequences to different taxonomy levels. Sequences were clustered into operational taxonomic units (OTUs).

### 2.9. Isotope Calculations

Stable carbon isotope ratios were expressed as δ^13^C values relative to Vienna Pee Dee Belemnite (VPDB) according to international standards, as in Equation (1) [53,54].

(1) $$\delta =\frac{R_{\mathrm{sample}}-R_{\mathrm{standard}}}{R_{\mathrm{standard}}}\times 1000‰$$

Benzene and toluene with heavy and light isotopes are degraded at slightly different rates, reflecting the kinetic isotope effects. The carbon isotope value of residual benzene and toluene, the non-degraded fraction of pollutant molecules, changes according to the Rayleigh equation [55,56]:

(2) $$\ln\left(\frac{1000+\delta_{t}}{1000+\delta_{0}}\right)=(\alpha -1)\times \ln f=\frac{\varepsilon }{1000}\times \ln f$$

(3) $$f=\frac{c}{c_{0}}$$

where *f* is the fraction of non-degraded benzene or toluene, *δ_t_* is the corresponding carbon isotope value, and *δ*_0_ is the carbon isotope value of benzene or toluene at the beginning of the degradation (*f* = 1). In Equation (2), *ε* is the isotope enrichment factor, which indicates the change in the isotope ratios according to the extent of transformation. A value of *ε* > 0 indicates a normal isotope effect, whereby benzene or toluene with light isotopes is degraded preferentially. As for *ε* < 0, the preferential degradation of heavy isotopes results in an inverse isotope effect. In Equation (3), *c* is the corresponding concentration and *c*_0_ is the concentration of benzene or toluene at the beginning of the degradation.

The actual biodegradation rates of benzene and toluene were calculated according to Equation (4), using the ‘*ε*’ value from the batch experiment and the δ^13^C value of the bio-PRB, where *B* is the actual biodegradation rate in the bio-PRB system [57].

(4) $$B=(1-f)\times 100\%=\left[1-{\left(\frac{1000+\delta }{1000+\delta_{0}}\right)}^{1000/\varepsilon }\right]\times 100\%$$

## 3. Results and Discussion

### 3.1. Apparent Removal Rate of Benzene and Toluene in Bio-PRB System

**Figure 4:**
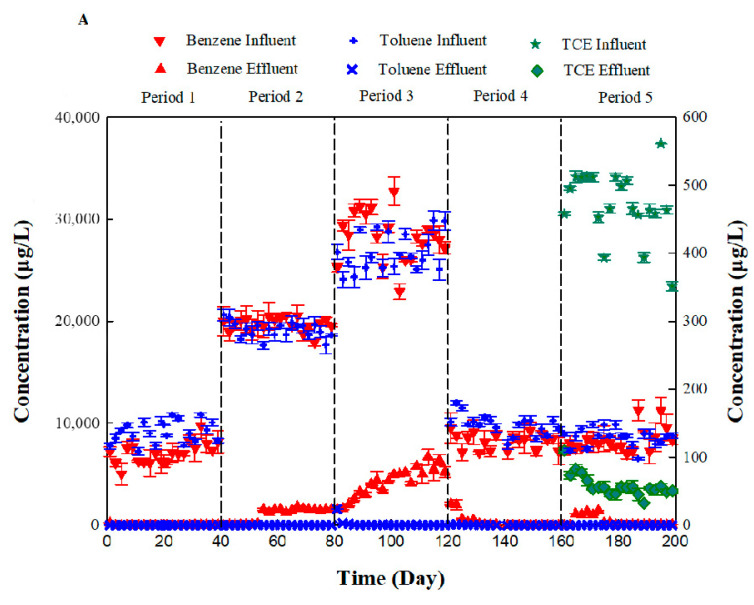

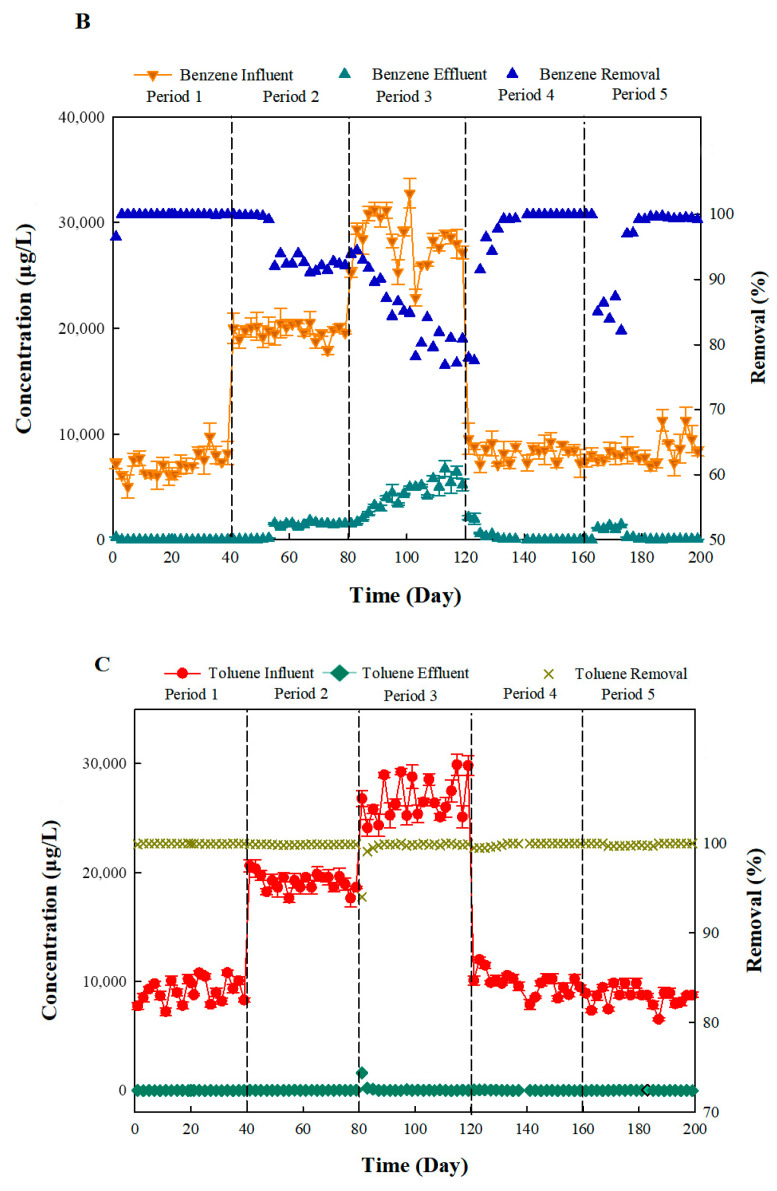
(**A**) Influent and effluent concentration of benzene and toluene in different periods. (**B**) Apparent removal rate of benzene. (**C**) Apparent removal rate of toluene. Apparent removal rate = effluent concentration/influent concentration.

Although benzene and toluene (both at an influent concentration of 10 mg L^−1^) replaced glucose as the carbon source in period 1 (Figure 4), the bio-PRB system could still effectively remove benzene and toluene from the synthetic groundwater. In periods 2–3, the removal rate of benzene decreased when the influent concentration increased from 10 mg L^−1^ to 30 mg L^−1^, suggesting a stress effect on the bio-PRB. However, when the influent concentration of benzene and toluene was reset to 10 mg L^−1^, the apparent removal rate of benzene was restored. The addition of TCE slightly inhibited the apparent removal rate of benzene (Figure 4A). In contrast to benzene, toluene could be effectively removed by the bio-PRB even when the influent concentration increased to 30 mg L^−1^, suggesting that an increase in the concentration of benzene and toluene did not cause an obvious stress effect on the apparent removal rate of toluene in the bio-PRB. Furthermore, although previous studies have reported that TCE had an inhibitory effect on the biodegradation of BTEX [26,58,59,60], our present work indicated that addition of TCE had little impact on the apparent removal rate of toluene. Thus, if we were to evaluate the influence of stress conditions on the bio-PRB based only on the apparent removal rate of BTEX, we would probably draw the wrong conclusions. On the other hand, more than 50% of the TCE was removed by the bio-PRB in period 5, which suggested the cometabolic biodegradation of benzene, toluene, and TCE in the bio-PRB. This cometabolism is discussed further in Section 3.5 and Section 3.6.

### 3.2. Batch Biodegradation Kinetics

**Figure 5:**
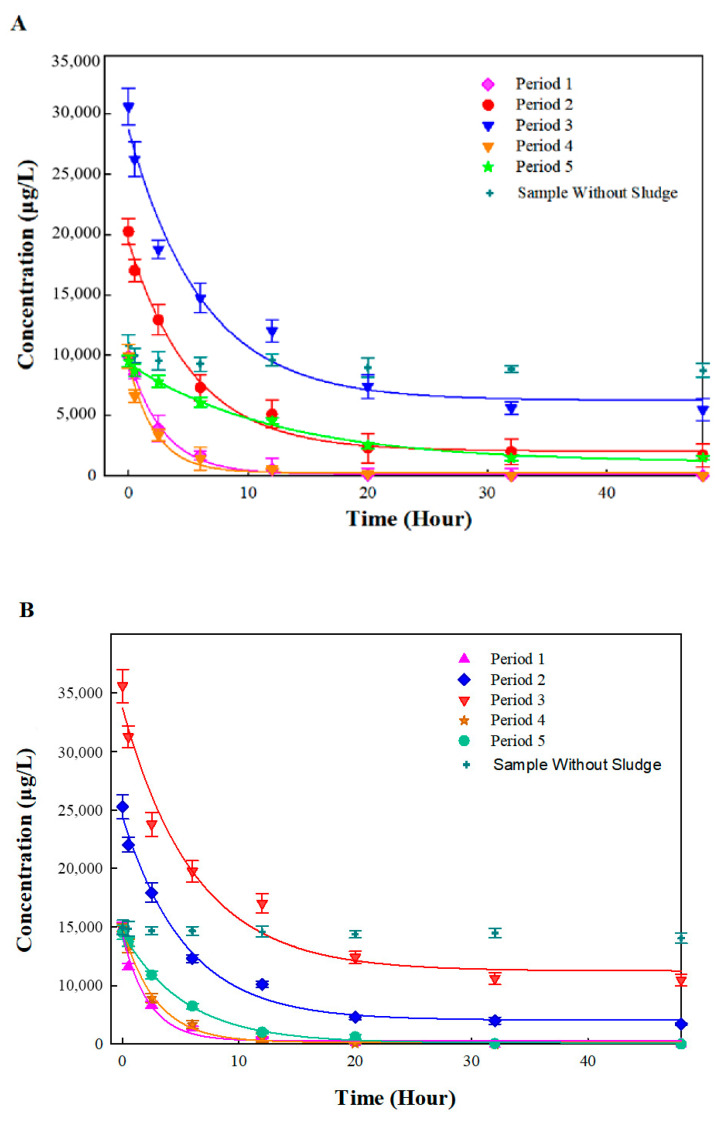
First-order biodegradation rate constants for benzene (**A**) and toluene (**B**) in different periods.

Batch experiments corresponding to five stages in the bio-PRB process were conducted to elucidate the biodegradation kinetics. In contrast to the apparent removal rates in the bio-PRB, the biodegradation kinetic rate of benzene and toluene in the batch experiments decreased under stress conditions (Figure 5). The benzene and toluene biodegradation rate constants in each period were obtained according to the pseudo-first-order equation and ranged from 0.0809 h^−1^ to 0.4468 h^−1^ and 0.1020 h^−1^ to 0.4618 h^−1^, respectively (Figure 5). In period 1, the bio-PRB demonstrated high biodegradation rate constants for benzene and toluene. With an increased influent concentration (periods 2 and 3), the benzene and toluene biodegradation rate constants decreased, suggesting an inhibition of the microbial activity. However, when the influent concentration of benzene and toluene decreased to 10 mg L^−1^ in period 4, the biodegradation rate constants and half-lives were almost restored, indicating the recovery of the microbial activity. Notably, when TCE (0.5 mg L^−1^) was added in period 5, not only did the benzene and toluene biodegradation rate constants decrease, but the half-lives of the benzene and toluene were prolonged. Hence, although the bio-PRB maintained a high apparent removal rate of benzene and toluene under stress conditions (Figure 4), the biodegradation kinetics of benzene and toluene were seriously inhibited. This conclusion is further discussed and confirmed in the following section.

### 3.3. Compound-Specific Isotope Analysis

**Figure 6:**
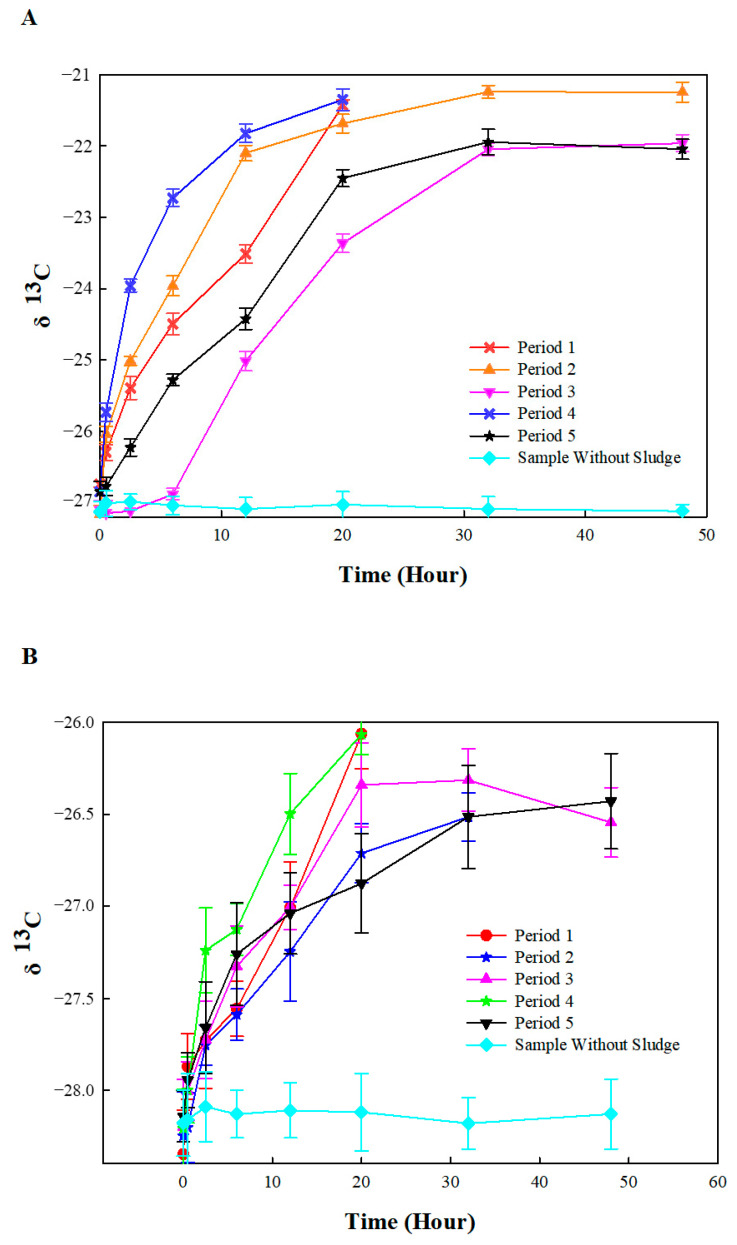
Variation in δ^13^C value of benzene (**A**) and toluene (**B**) in different periods.

Microorganisms preferentially use lighter isotope species due to the lower energy cost, resulting in fractionations between heavier and lighter isotopes. Thus, positive shifts in the ratio of ^13^C to ^12^C (higher δ^13^C values) in organic pollutants could represent the biodegradation rate [44,53]. According to this method, we evaluated the biodegradation rate of benzene and toluene based on the shifts in the isotopic composition in each period (Figure 6). The δ^13^C value of both benzene and toluene in all periods changed over time, namely Δ^13^C = +4.8‰~+6.0‰ VPDB for benzene and +1.7‰~+2.7‰ VPDB for toluene, which represented the microbial activity for the biodegradation of benzene and toluene. 

**Figure 7:**
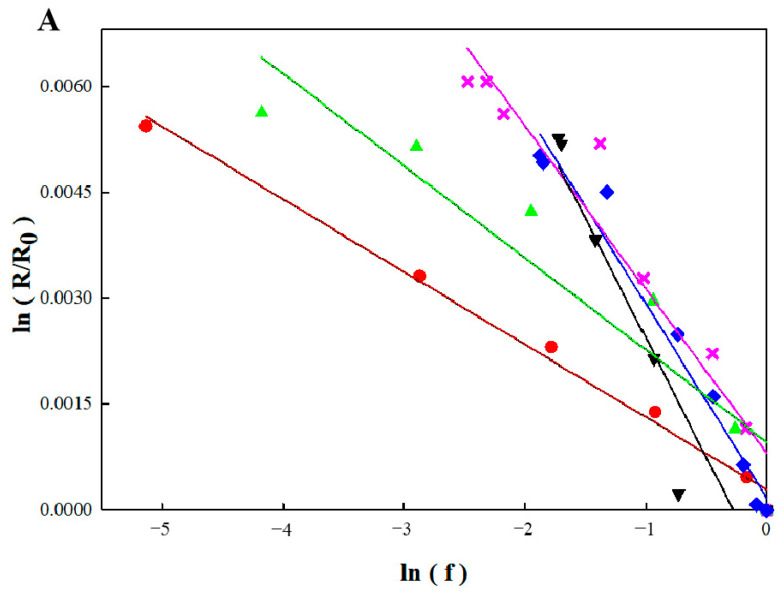

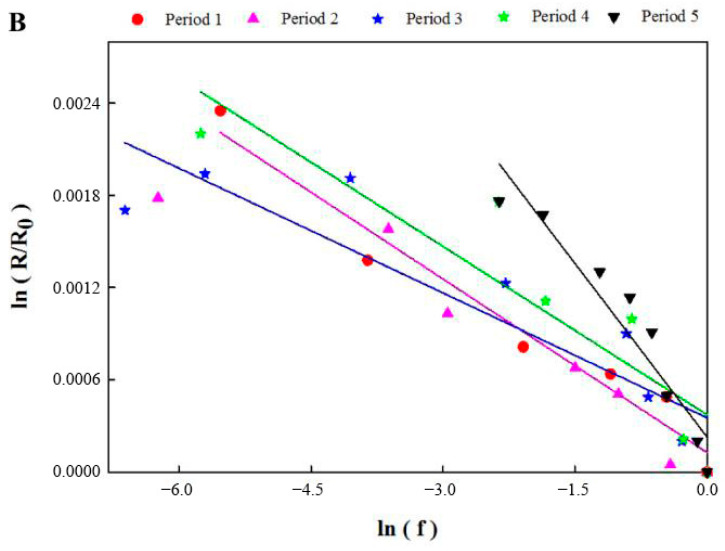
Rayleigh plots for carbon isotope fraction of benzene (**A**) and toluene (**B**) biodegradation.

In order to illustrate the correlation between the initial organic pollutant (benzene or toluene) concentration and the isotope ratios, these parameters were plotted according to the Rayleigh equation (Equation (2)). Data pertaining to the benzene and toluene from each individual experimental period were fit using the Rayleigh model (Figure 7) with *R*^2^ = 0.8878~0.9922 and 0.9061~0.9803, respectively (Table 1), which indicated that the Rayleigh enrichment factors (ε_c_) of benzene (−3.4‰ to −1.0‰) were influenced not only by the influent concentration but also by the addition of TCE. The ε_c_ values of benzene were −1.0‰, −2.3‰, and −3.4‰ in periods 1, 2, and 3, respectively, indicating that benzene biodegradation was inhibited by an increase in the influent concentration. However, in period 4, the ε_c_ of benzene increased to −1.3‰ as the influent concentration was reduced to 10 mg L^−1^, suggesting that the benzene biodegradation activity recovered. In addition, due to the inhibition caused by TCE, the åc of benzene decreased to −2.8‰ again in period 5. 

Previous research has reported that variation in the microbial community in the bio-PRB led to different enrichment factors in each period. Thus, microorganisms belonging to different phylogenetic groups generated different extents of stable isotopic fractionation due to the influence of processes such as uptake into the cell, transport across the membrane, and binding to the enzyme in the bio-PRB [44,61]. Our previous laboratory studies with anaerobic benzene biodegradation cultures showed pronounced carbon isotope fractionation under sulfate-reducing (ε_c_ = −3.6‰) and methanogenic conditions (ε_c_ = −2.1‰~1.9‰) [44,47]. The variation in the carbon isotope fractions of benzene in different periods suggested that the total number of methanogenic bacteria decreased, while sulfate-reducing bacteria gradually grew to be the dominant population with the continuous increase in the influent concentration. Meanwhile, the decrease in the enrichment factors in period 5 indicated that the sulfate-reducing bacteria had stronger tolerance than methanogenic bacteria under the TCE stress conditions.

In contrast to benzene, the enrichment factors of toluene were not influenced by the influent concentrations, although a slight variation in the ε_c_ (−0.4‰ to −0.3‰) caused by a statistical error was observed in period 3 [61]. Previous studies have also reported only a slight variation in the carbon isotope fractionation of toluene via the anaerobic pathway involving monooxygenase or dioxygenase [62]. In addition, the slight variation in the ε_c_ value (−0.4‰ to −0.8‰) observed in period 5 also led to the serious inhibition of the toluene biodegradation. On the other hand, although the rate limitation in the transition state of the bond cleavage and the nature of the chemical reaction could influence the extent of the kinetic isotope effect, complex enzyme-catalyzed reactions are the rate-limiting steps that affect the ‘‘apparent” isotope [63].

### 3.4. Calculation of Actual Biodegradation Rates of Benzene and Toluene in Bio-PRB

In a bio-PRB, BTEX can be removed by biodegradation and physical separation (e.g., dilution, dispersion, volatilization, and sorption). Biodegradation is the process in which BTEX are degraded by microorganisms, while the latter is the transfer process whereby BTEX are likely to leak into the groundwater again after a certain period of time, leading to secondary pollution. Since the adsorption of benzene and toluene cannot influence their carbon isotope fractionations, the actual biodegradation rates of benzene and toluene in different periods could be calculated by the carbon isotope enrichment factors obtained from the batch experiments and the δ^13^C value of the bio-PRB (Table 2) calculated according to Equation (3). The actual biodegradation rate of benzene decreased from 99.62% to 77.81% as the influent concentration increased to 30 mg L^−1^ in period 3 and, subsequently, was restored to 98.74% when the influent concentration returned to 10 mg L^−1^ in period 4. This indicated that although an increasing benzene and toluene influent concentration had a stress effect on the biodegradation of benzene, this inhibition almost disappeared as the influent concentration decreased to the initial level. On the contrary, the actual biodegradation rate of toluene remained stable during periods 1–4, which indicated that increasing the influent concentration of benzene and toluene did not have an obvious inhibitory effect on the toluene biodegradation. In addition, the actual biodegradation rates of benzene and toluene decreased from 98.74% to 80.26% and from 99.27% to 85.75%, respectively, in period 5, which indicated that both benzene and toluene biodegradation were inhibited by TCE in the bio-PRB.

It was noteworthy that the apparent removal rates of benzene and toluene were much higher than their actual biodegradation rates, as calculated by the carbon isotope enrichment factors of the batch experiments and the δ^13^C value of the bio-PRB. This significant difference may be explained as follows: On the one hand, the active microorganisms were inhibited under stress conditions, resulting in a decrease in the actual biodegradation rates of benzene and toluene. On the other hand, although the biofilm of the bio-PRB aged and then detached from the filler as the operation proceeded, the detachment of the aging biofilm from the porcelain granules led to the exposure of a greater surface area and more pore channels, increasing the adsorption of benzene and toluene to a certain degree, which could have offset the inhibition of active microorganisms under stress conditions (Figure S2). Thus, according to the above results, both the apparent removal rate and biodegradation rate of organic pollutants should be determined in order to accurately evaluate the biodegradation of organic pollutants in bio-PRBs.

### 3.5. Functional Gene Copies in Bio-PRB System

**Figure 8:**
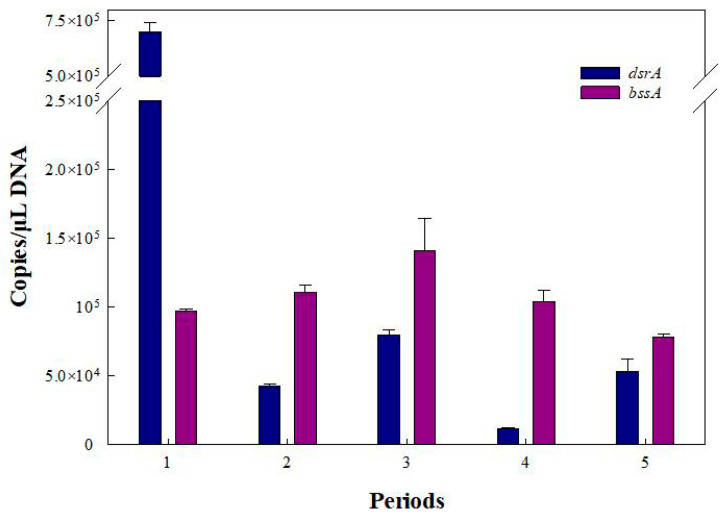
Absolute abundance of *dsrA* and *bssA* genes in bio-PRB system in different periods.

Benzylsuccinate synthase (*bssA*) has frequently been found in denitrifying, sulfate-reducing, and methanogenic bacteria that are responsible for toluene biodegradation [49]. In addition, dissimilatory sulfite reductase (*dsrA*) is a key enzyme for sulfate reduction in all sulfate-reducing bacteria that it is used as a biomarker to directly analyze the populations of these bacteria [64]. In order to further elucidate the benzene and toluene biodegradation mechanisms, the abundance of related functional genes (*bssA*, *dsrA*) was monitored using q-PCR analysis. Due to the induction of toluene, the abundance of *bssA* increased with the influent concentration of benzene and toluene in periods 1–3 (Figure 8). The high amount of *bssA* in period 4 was a remnant of the high level in period 3. The increase in the influent concentration could stimulate the expression of *dsrA*, showing a significant correlation with *dsrA* copies [65]. However, the high concentration of the carbon source (glucose: 300 mg L^−1^) used in the start-up period led to a high amount of *dsrA* copies in the bio-PRB, which caused the *dsrA* level to remain high in period 1. Thus, the increase in the influent concentrations could have stimulated the microbes in the bio-PRB so as to increase the related functional genes (*bssA* and *dsrA*), which is consistent with previous studies [66]. Notably, the number of *dsrA* copies increased while the number of *bssA* copies decreased under TCE stress in period 5, indicating a cometabolic effect involving benzene, toluene, and TCE. Previous research has also reported similar cometabolic biodegradation in the biodegradation of PCBs. Thus, the co-metabolic biodegradation of TCE, benzene, and toluene by *dsrA* probably in turn stimulated and increased the amount of *dsrA*. 

Among the functional genes tested in this study, *bssA* had a higher abundance in periods 2–5 (Figure 8). The maximum difference between *dsrA* and *bssA* in period 4 suggested that methanogenic bacteria became the dominant population. Moreover, the minimal difference between *dsrA* and *bssA* in period 5 suggested that sulfate-reducing bacteria gradually grew to be the dominant population and that the methanogenic process was inhibited. The results were consistent with the analysis of the carbon isotope values in all periods. 

### 3.6. Bacterial Community Analyses

**Figure 9:**
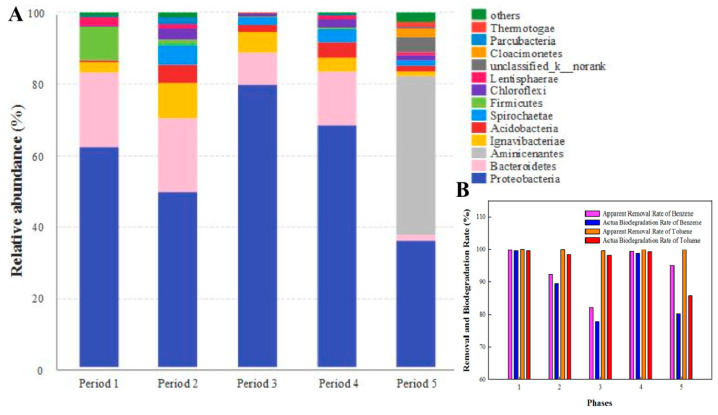
(**A**) Taxonomic classification of the microbial community in the bio-PRB system at phylum level. (**B**) Apparent removal rate and actual biodegradation rate of benzene and toluene.

**Figure 10:**
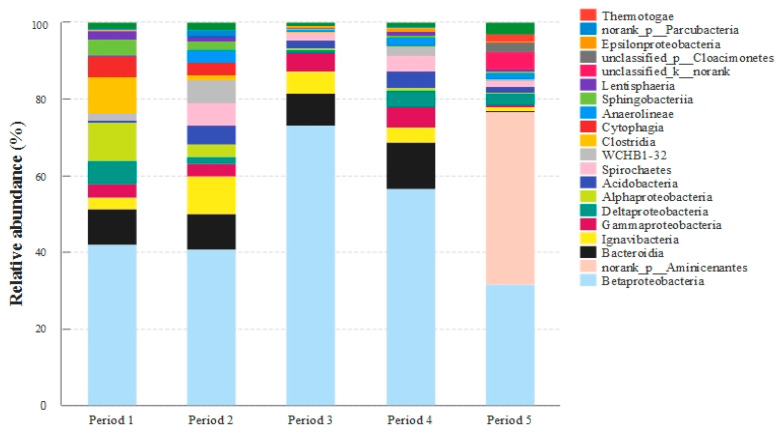
Taxonomic classification of the bacterial community in the bio-PRB at class level.

**Figure 11:**
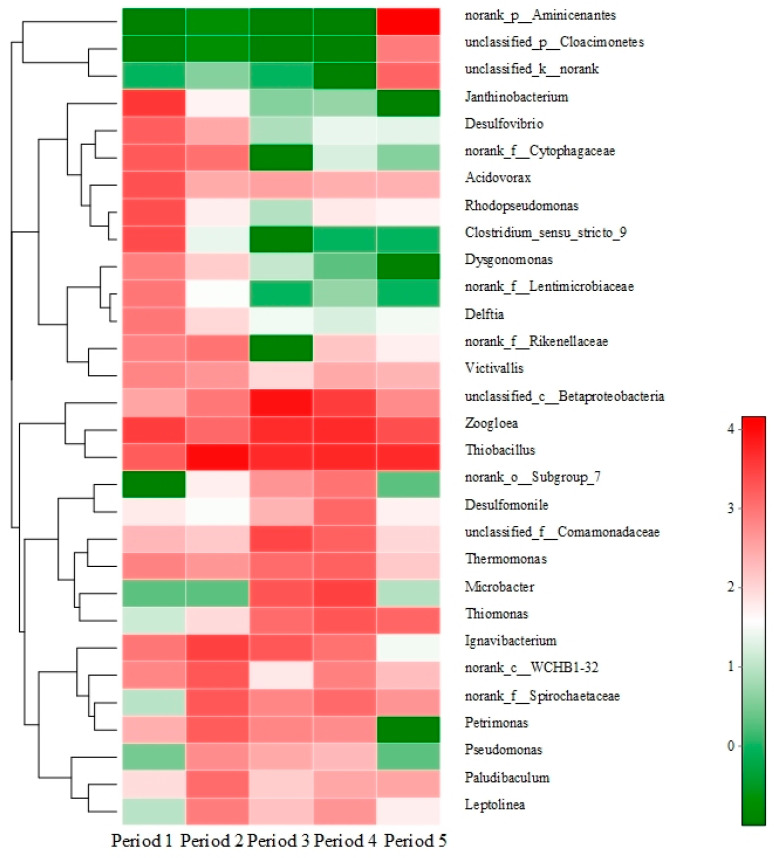
Taxonomic classification of the bacterial community in the bio-PRB at genus level.

To further explore the influence of stress conditions on the microbial community, the microbiocenoses in the bio-PRB were analyzed. All raw reads were generated from the Illumina high-throughput sequencing Mi-Seq platform. After quality filtration, all data sets were randomly subsampled to an equal depth of 35128 OTUs. The rarefaction curves indicated an adequate level of sequence coverage for the total community analysis of all periods (Figure S1). The OTU classification data of the total DNA samples were used to determine the bacterial community composition in each of the five samples. The bacterial communities corresponding to the sequences of the total genomic DNA samples (relative abundance >1%) are shown in Figure 9, Figure 10 and Figure 11.

It was observed that the bacterial community of the bio-PRB varied across different periods. Besides the small percentage of unclassified bacteria, *Proteobacteria* were the most abundant (relative abundance of 35.9–79.7%) phylum, followed by *Bacteroidetes* (1.51–20.9%) and *Ignavibacteriae* (1.15–9.82%) (Figure 9A). Since *Ignavibacteriae*, *Bacteroidetes*, *Aminicenantes*, and *Proteobacteria* are frequently found in BTEX-impacted environments [24,66,67,68], the present work focused on these four dominant phyla. The relative abundance of *Proteobacteria* increased with the influent concentration of benzene and toluene, which indicated that *Proteobacteria* had a strong tolerance to a high concentration of benzene and toluene. Moreover, *Betaproteobacteria* was the most dominant class in the bio-PRB, and its relative abundance was positively correlated to the influent concentration of benzene and toluene (Figure 10). It has been reported that increasing the influent concentration of benzene and toluene in an anaerobic environment could lead to the acidification of the bio-PRB [24]. Thus, with its strong tolerance to acidic environments, *Betaproteobacteria* is responsible for the biodegradation of benzene and toluene even at a high influent concentration. In addition, at the genus level, the relative abundance of *unclassified_c_Betaprobeobacteria* and *Ignavibacterium* increased with the influent concentration, showing a strong tolerance to a high concentration of benzene and toluene (Figure 11). Notably, the abundance of these dominant bacteria communities (*Proteobacteria* and *Bacteroidetes*) in period 4 was similar to that in period 1, suggesting the restoration of microbe communities. This inference is also verified by the high biodegradation rate of benzene and toluene in period 4 (Figure 9B). 

The addition of TCE not only inhibited actual biodegradation (Figure 9B) but also caused changes in the bacterial community (increase in species diversity) in the bio-PRB (Table S5). The dominant bacterial community (*Ignavibacteriae* and *Bacteroidetes* at the phylum level, *Ignavibacteria* and *Bacteroidia* at the class level) varied significantly. The newly appeared *Aminicenantes*, responsible for the biodegradation of TCE, became the most dominant phylum (relative abundance = 44.6%) [67], while the relative abundance of *Proteobacteria* (35.9%), *Bacteroidetes* (1.53%), and *Ignavibacteriae* (2.91%) drastically decreased. *Aminicenantes* are anaerobic bacteria that can be found in hydrocarbon-polluted environments. Due to its high intraphylum metabolic diversity and adaptive capabilities, *Aminicenantes* can survive in a wide range of pH and environmental conditions [68]. Thus, *Aminicenantes* alleviated the TCE stress in the bio-PRB and played a primary role in the biodegradation of TCE, benzene, and toluene. Furthermore, at the genus level (Figure 11), *unclassified_c_Betaprobeobacteria)* and *Ignavibacterium* decreased significantly, suggesting their inhibition by TCE stress. Although the syntrophic acetogenic bacteria *Petrimonas* is responsible for BTEX biodegradation via the production of electron donors (hydrogen) for TCE degradation [69], the decrease in the relative abundance of *Petrimonas* inhibited the hydrogen production under TCE stress. On the contrary, *norank_p_Aminicenantes*, which appeared with a high abundance, played an important role in the bio-PRB under TCE stress. Although *Acidovorax* has been described as capable of degrading BTEX [24,67,68], its poor tolerance to TCE stress meant that it hardly contributed to BTEX biodegradation in our experiment.

**Figure 12:**
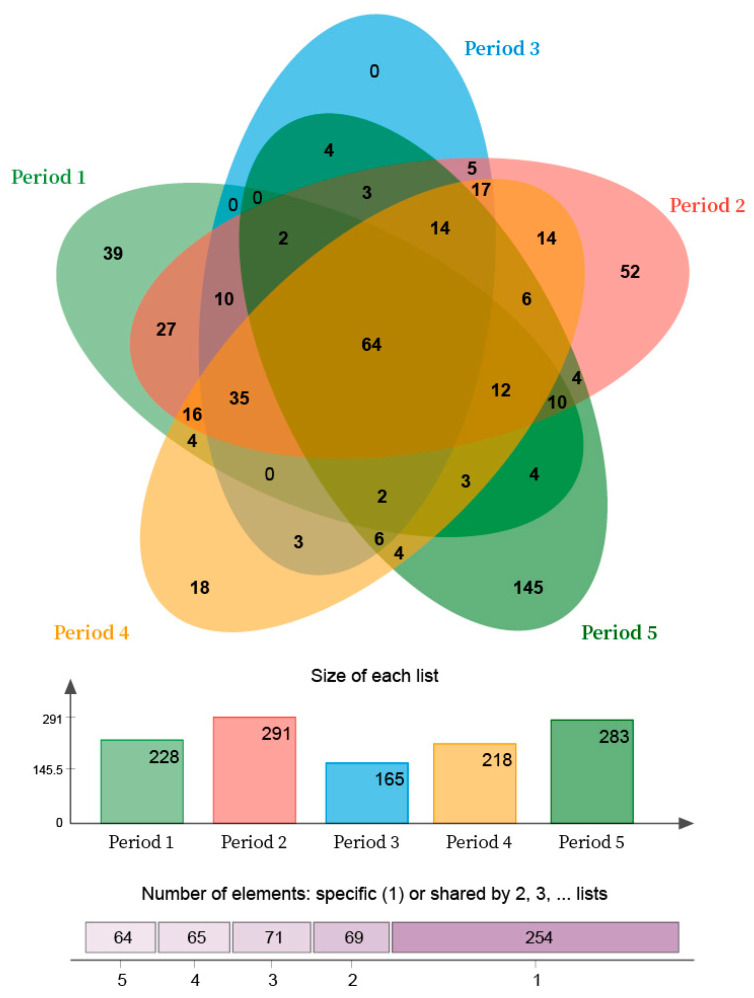
Venn map of bio-PRB system in all periods.

The Venn map of the bio-PRB system indicated that 523 unique OTUs were detected across the five periods (Figure 12). Among them, 64 OTUs were detected in all periods. The number of unique OTUs in periods 2, 3, and 5 was 52, 0, and 145, respectively, suggesting that the addition of TCE influenced bacterial community diversity more substantially than an increasing influent concentration. Furthermore, the number of OTUs with similar genes was 166 in periods 1 and 2, 113 in periods 1 and 3, 135 in periods 1 and 4, respectively. Thus, although the bacterial community was influenced by the increasing influent concentration in periods 2 and 3, it could still be restored to the level of period 1 as the influent concentration decreased to 10 mg L^−1^. In addition, the number of OTUs with similar genes in periods 1 and 5 was 97, also confirming the more substantial influence of TCE on the bacteria community. On the other hand, due to their self-regulation ability, the number of OTUs in the bio-PRB increased in order to adapt to stress conditions.

### 3.7. Conceptual Process of Benzene and Toluene Removal

**Figure 13:**
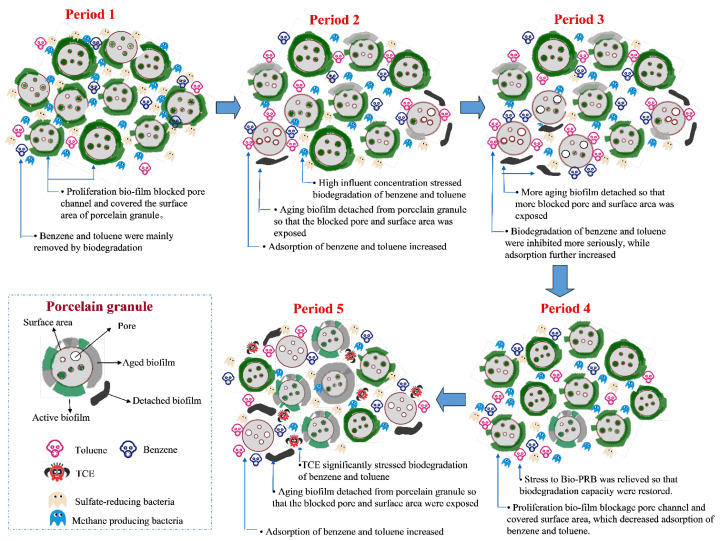
Scheme of benzene and toluene removal by bio-PRB in all periods.

Based on the above analysis and results, we proposed a conceptualization of the benzene and toluene removal process in the bio-PRB (Figure 13). Generally, benzene and toluene can be removed by adsorption (porcelain granules and biofilm) and biodegradation (active biofilm and suspended microorganisms). In period 1, the pore channels and surface area of the porcelain granules were blocked by the biofilm proliferation, resulting in a decrease in their adsorption capacity. Thus, benzene and toluene were mainly removed by biodegradation. In periods 2 and 3, with the inhibition caused by the high influent concentration, the biodegradation of benzene and toluene decreased. However, the greater pore and surface area exposure due to the detachment of the aging biofilm from the porcelain granules likely increased their benzene and toluene adsorption capacity, and so the apparent removal rate of the chemicals did not decrease as much as their actual biodegradation rate (Table 2). As for period 4, although the microbial community and biodegradation capacity were restored under non-stress conditions, the blockage of the pore channels and the coverage of the surface area caused by the biofilm proliferation decreased the adsorption capacity of the porcelain granules. Thus, benzene and toluene were mainly removed by biodegradation processes in period 4. In period 5, the addition of TCE inhibited the microbial activity, with the biofilm detached from the porcelain granules, resulting in greater surface area and pore channel exposure for the adsorption of benzene and toluene. Thus, the inhibition of the biodegradation activity was probably offset by the enhancement in the adsorption capacity to a certain degree, which meant that the apparent removal rate did not decrease substantially. In addition, due to their stronger tolerance to TCE stress compared with methanogenic bacteria, sulfate-reducing bacteria played a primary role in the biodegradation of benzene and toluene in period 5.

## 4. Conclusions

By using compound-specific isotope analysis in combination with high-throughput sequencing analysis, we successfully evaluated BTEX biodegradation in a bio-PRB and the stress response of the microbe community. A greater decline in the biodegradation rate of benzene and toluene was observed compared with the apparent removal rate under stress conditions. Both the addition of TCE and the increase in the influent concentration significantly decreased the biodegradation rate of benzene, while the toluene biodegradation rate was only inhibited by TCE. Although the relative abundance of the dominant bacterial community responsible for the biodegradation of benzene and toluene decreased as the influent concentration increased, it was almost completely restored as the influent concentration decreased. Compared with the increasing influent concentration, the addition of TCE had a greater influence on the bacterial community. In the bio-PRB, *Betaproteobacteria* was the dominant class for the biodegradation of benzene and toluene. After adding TCE, *Aminicenantes* became the most dominant phylum, as it could cometabolize TCE, benzene, and toluene, while the other members of the bacterial community were seriously inhibited. Due to its poor tolerance to stress conditions, the contribution of *Acidovorax* to BTEX biodegradation was limited. The increase in *Aminicenantes* and the decrease in *Acidovorax* suggested the existence of an antagonistic relationship between them.

## Figures and Tables

**Table 1:** Enrichment factors (ε_c_) of benzene and toluene biodegradation in different periods (n = 3).

Period	ε_c_ of Benzene (‰)	*R* ^2^	ε_c_ of Toluene (‰)	*R* ^2^
1	−1.0	0.9922	−0.4	0.9615
2	−2.3	0.9372	−0.4	0.9803
3	−3.4	0.9099	−0.3	0.9061
4	−1.3	0.8878	−0.4	0.9062
5	−2.8	0.9710	−0.8	0.9559

**Table 2:** Actual biodegradation rate and apparent removal rate of benzene and toluene in different periods.

Period	δ^13^C of Remaining Benzene	Actual Biodegradation Rate of Benzene (%)	Apparent Removal Rate of Benzene (%)	δ^13^C of Remaining Toluene	Actual Biodegradation Rate of Toluene (%)	Apparent Removal Rate of Toluene (%)
1	−21.4689	99.62	99.83	−26.0785	99.59	99.97
2	−22.0532	89.40	92.37	−26.5468	98.37	99.88
3	−22.1076	77.81	82.00	−27.2187	98.10	99.55
4	−21.2965	98.74	99.36	−26.3108	99.27	99.85
5	−22.4165	80.26	95.18	−26.6258	85.75	99.89

## Data Availability

The data presented in this study are openly available.

## Supplementary Materials

The following are available online at https://www.mdpi.com/article/10.3390/ijerph19148800/s1, S1.1: Gas chromatography analysis, S1.2: CSIA analysis, S1.3: DNA extraction and PCR amplification, S1.4: Illumina high-throughput sequencing, S1.5: Extraction of EPS, S1.6: SEM analysis, Figure S1: The relationship between δ^13^C values and signal intensity, Figure S2: Standard curve of contaminant concentration–signal intensity, Figure S3: EPS of bio-PRB in all periods, Figure S4: Variation of biofilm in all periods, Figure S5: Rarefaction curves of the five activated sludge samples at cutoff level of 3% (the rarefaction curve, plotting the number of observed OTUs as a function of the number of sequences, was computed using RDP’s pyrosequencing pipeline), Table S1: Components of the synthetic groundwater used in the bio-PRB system, Table S2: Operational parameters of the bio-PRB system, Table S3: Batch experimental design for the removal of benzene and toluene, Table S4: The measuring accuracy of benzene and toluene for δ^13^C values, Table S5: Sequences of primers of functional genes, Table S6: Diversity index (3% cutoff). References [45,46,47,48,49,50,51,52] are cited in the Supplementary Materials.
