# Supplementary material for: Assessing the Biodegradation of BTEX and Stress Response in a Bio-Permeable Reactive Barrier Using Compound-Specific Isotope Analysis
Source: Int J Environ Res Public Health. 2022 Jul 20;19(14):8800. doi: 10.3390/ijerph19148800 (PMC9322891; doi:10.3390/ijerph19148800)
Supplement: Supplementary file 1 [file ijerph-19-08800-s001.zip › ijerph-1622755-supplementary.pdf]

Supplementary materials

S1. Methods

S1.1 Gas chromatography analysis

Sample (5 mL) was introduced into the purging vessel with a syringe [1]. The sample was purged with helium gas at 35 mL min^{-1} for 11 min. The purged volatile compounds were trapped on a Tenax trap. The trap was heated to 225°C and kept at the same temperature for 2 min to desorb benzene and toluene which were then applied to the GC column. The operating parameters and flow rates were as follows: the injector and detector temperatures were set at 280°C and 220°C , respectively. The split ratio was 5:1. The oven temperature was held at 30°C for 3 min then programmed at 15°C min^{-1} to 180°C.

[1] G.A. E, C.L. S, E.A. D, Standard methods for the examination of water and wastewater., Sci. Total Environ. 142 (1992) 227-228.

S1.2 CSIA analysis

Benzene and toluene were extracted from samples by using a Solid Period Micro Extraction (SPME) fiber at 25°C. Benzene and toluene working standards calibrated against International Reference Material for carbon isotope were prepared and analyzed using the same method as for real samples to correct for any isotope fractionation occurring during SPME extraction. Benzene and toluene were desorbed from SPME fiber in GC inlet at 270°C then separated on the GC column. The GC temperature program was 40°C held for 3 min, then increased at 15°C min^{-1} to 180°C. The helium carrier gas flow through the column was 1.5 mL min^{-1}. The oxidation reactor for $^{13}C/^{12}C$ was set at 940°C. The pulse of the reference gas (CO_2, $\delta^{13}C$: -26.42‰ VPDB) were used for the computation of the isotopic values of sample compounds. Benzene and toluene standards were analyzed repeatedly three times under the same operating conditions, and the standard deviation of the measurements was typically within ±0.5‰ [2, 3].

[2] S.A. Mancini, A.C. Ulrich, G. Lacrampe-Couloume, B. Sleep, E.A. Edwards, B.S. Lollar, Carbon and hydrogen isotopic fractionation during anaerobic biodegradation of benzene, Appl. Environ. Microbiol. 69 (2003) 191-198.

[3] A. Fischer, J. Bauer, R.U. Meckenstock, W. Stichler, C. Griebler, P. Maloszewski, M. Kästner, H.H. Richnow, A multitracer test proving the reliability of Rayleigh equation-based approach for assessing biodegradation in a BTEX contaminated aquifer, Environ. Sci. Technol. 40 (2006) 4245-4252.

S1.3 DNA extraction and PCR amplification

Polymerase chain reaction (PCR) amplification was carried out in 30 μL reaction volume containing 2 μL template DNA, 100 mM dNTP, 1 PCR buffer, 1 U of EXTaq polymerase (TransGen Biotech, China) and 2 mM each primer set (Table S4). Thermal cycling consisted of initial denaturation for 5 min at 94°C, then 40 cycles: denaturation of 95°C for 30 s, annealing 55°C for 45 s and elongation 72°C for 50 s, followed by a 5 min extension at 72°C.

Real-time PCR was performed for all samples using oligonucleotides that are designed to target *dsrA* and *bss*A. The PCR primer sets like *dsrA*f - *dsrA*r and *bssA*f - *bssA*r were specific for genes *dsrA* and *bssA* [4-6]. The q-PCR mixture (20 mL) contained 10 mL of SYBR Premix EXTaq Super Mix (TaKaRa Japan), 0.3 mL of each primer set (10 mM), 8 mL of template DNA (5 ng/mL) and 1.4 mL of distilled H_2O (ddH_2O). The real-time PCR program was performed as follows: the reactions were run for 50 cycles, and initial denaturation for 10 min at 95°C, then denaturation of 95°C for 15 s, annealing for 1 min at 55°C and elongation 72°C for 20 s. According to Rhee, DNA cloning was used to construct recombinant plasmids carrying *dsrA and bssA,* and five to seven-point calibration curves (C_t values versus log of initial target gene copy) were generated for the q-PCRs using 10-fold serial dilution of the plasmid [7].In order to take into account the variation of DNA extraction efficiency of samples, the relative abundance of each target gene was normalized to eubacterial 16S rRNA gene [8]. The reaction efficiency of dsrA was 85.2% and bssA was 104.7% with R^2 values more than 0.995 for all calibration curves.

[4] H.R. Beller, S.R. Kane, T.C. Legler, P.J. Alvarez, A real-time polymerase chain reaction method for monitoring anaerobic, hydrocarbon-degrading bacteria based on a catabolic gene, Environ. Sci. Technol. 36 (2002) 3977-3984.

[5] J.R. Stults, O. Snoeyenbos-West, B. Methe, D.R. Lovley, D.P. Chandler, Application of the 5' fluorogenic exonuclease assay (TaqMan) for quantitative ribosomal DNA and rRNA analysis in sediments, Appl. Environ. Microbiol. 67 (2001) 2781-2789.

[6] B.R. Baldwin, A.D. Peacock, M. Park, D.M. Ogles, J.D. Istok, J.P. Mckinley, C.T. Resch, D.C. White, Multilevel samplers as microcosms to assess microbial response to biostimulation, GroundWater. 46 (2008) 295-304.

[7] S.K. Rhee, X. Liu, L. Wu, S.C. Chong, X. Wan, J. Zhou, Detection of genes involved in biodegradation and biotransformation in microbial communities by using 50-mer oligonucleotide microarrays, Appl. Environ. Microbiol. 70 (2004) 4303-4317.

[8] J.C. López-Gutiérrez, S. Henry, S. Hallet, F. Martin-Laurent, G. Catroux, L. Philippot, Quantification of a novel group of nitrate-reducing bacteria in the environment by real-time PCR, J. Microbiol. Meth. 57 (2004) 399-407.

S1.4 Illumina High-through sequencing

Raw sequences were generated by the sequencing strategy of Index 101 PE (Paired-End sequencing, 101-bp reads and 8-bp index sequence). The quality control (QC) pipeline was applied to remove the adaptor at the end of reads and unknown nucleotides of the raw sequences were firstly removed. And then Galaxy (http://usegalaxy.org/)was used to conduct a stricter filtration. In order to ensure each filtered read possessed Illumina greater quality, quality formats were converted and low quality sequences were removed.

The filtered Illumina reads of the five biofilm samples were processed and analyzed on http://www.i-sanger.com/. The results were subsampled to 35,128 sequences (*i.e.*, the number of sequences in the sample with the least number of sequences). The confidence threshold of 97% recommended by the RDP was applied to strictly assign the sequences to different taxonomy level. Sequences were clustered into operational taxonomic units (OTUs).

S1.5 Extraction of EPS

Biofilm samples were extracted using a heat extraction method.[1] A 10 mL of biofilm sample was used for base extraction. The samples were first centrifuged at 9000 rpm for 15 min to separate the liquid and solid, and pellets were resuspended with saline water (0.9% NaCl solution). The mixture was heated in 100 ℃ for 1 h washed, and then centrifuged at 3200 rpm for 30 min.(Extracellular polymeric substances diversity of biofilms grown under contrasted environmental conditions) The EPS was normalized as the sum of proteins and polysaccharides. Proteins were determined using the Folin methd with bovine serum albumin (BSA) as the standard. (Lowry, O.H., Protein measurement with the Folin phenol reagent) Polysaccharides were determined according to the phenol-sulfuric acid method with glucose as the standard. （New insight into influence of mechanical stirring on membrane fouling of membrane bioreactor: Mixed liquor properties and hydrodynamic conditions）

S1.6 SEM analysis

The surface morphologies was analyzed by scan electronic microscopy (SEM, S-3400N II, Hitachi, Japan) after preparation using the following procedure. Porcelain granules were fixed with 3% glutaraldehyde in PBS for 4 h, and then washed with PBS twice (5 min each), rinsed with deionised water and dehydrated with ethanol gradient (at 50%). The porcelain granules were freeze-dried in freeze dryer before SEM observation.

S2. Tables

Table S1. Components of the synthetic groundwater used in the Bio-PRB system

**Condition**	**Ingredients**	**Concentration (mg/L)**				
		**Period 1**	**Period 2**	**Period 3**	**Period 4**	**Period 5**
Synthetic groundwater	Benzene	10	20	30	10	10
	Toluene	10	20	20	10	10
	TCE	0	0	0	0	0.5
	$(NH_4)_2SO_4$	14.2	24.8	42.6	14.2	14.2
	K_2HPO_4	1.7	3.4	5.1	1.7	1.7
	KH_2PO_4	10.8	21.6	32.4	10.8	10.8
Trace elements solution	$CaCl_2$•$2H_2O$	2.4	2.4	2.4	2.4	2.4
	$MgSO_4$•$7H_2O$	16	16	16	16	16
	$FeSO_4$•$7H_2O$	1	1	1	1	1
	CuCl2•$2H_2O$	0.25	0.25	0.25	0.25	0.25
	$CoCl_2$•$6H_2O$	0.22	0.22	0.22	0.22	0.22
	$ZnSO_4$•$7H_2O$	1.01	1.01	1.01	1.01	1.01
	$MnCl_2$•$4H_2O$	1.22	1.22	1.22	1.22	1.22
	$NiCl_2$•$6H_2O$	0.02	0.02	0.02	0.02	0.02
	$(NH_4)_6MO_7O_{24}$•$4H_2O$	0.1	0.1	0.1	0.1	0.1

Table S2. Operational parameters of the Bio-PRB system

Periods	Duration (d)	Glucose concentration (mg/L)	Influent pH value	HRT (h)	Benzene Concentration (mg/L)	Toluene concentration (mg/L)	TCE concentration(mg/L)
Start-up period	1~30	300	8.50±0.10	48	0	0	0
1	1~40	0	8.50±0.10	48	10	10	0
2	41~80	0	8.50±0.10	48	20	20	0
3	81~120	0	8.50±0.10	48	30	30	0
4	121~160	0	8.50±0.10	48	10	10	0
5	161~200	0	8.50±0.10	48	10	10	0.5

Table S3. Batch experimental design for the removal of benzene and toluene

**Periods**	**pH**	**Sludge Volume (mL)**	**Benzene (mg/L)**	**Toluene (mg/L)**	**TCE (mg/L)**
1	8.50±0.10	50	10	10	0
2	8.50±0.10	50	20	20	0
3	8.50±0.10	50	30	30	0
4	8.50±0.10	50	10	10	0
5	8.50±0.10	50	10	10	0.5

Table S4. The measuring accuracy of benzene and toluene for δ13C values

Compounds	*Signal Intensity (mV)*	*Peaktime (min)*	*δ13C (‰)*	*Std. (n=3)*
Benzene	8217 ~ 9353	3.445 ~3.450	-27.12 ~ -26.72	0.20
Toluene	8137 ~ 8428	5.285 ~ 5.421	-28.23 ~ -28.01	0.11

Table S5. Sequences of primers of functional genes

Target	Sequence	Annealing Temp (℃)	Thermal profile for qPCR (40 Cycles)	Reference
dsrA	F:ACSCACTGGAAGCACG<br>R:TGCCGAGGAGAACGATGTC	53	95 ℃/10 min; 95 ℃/15 s,53 ℃/50 s,60 ℃/1 min	2-4
bssA	F:ACGACGGYGGCATTTCTC<br>R:GCATGATSGGYACCGACA	50	95 ℃/10 min; 95 ℃/15 s,50 ℃/50 s,60 ℃/1 min	2-4

Table S6. Diversity index (3% cutoff)

Periods	Sequence number	OTUs	Chao	Ace	Simpson
1	35128	183	268.2	287.7	0.04881
2	35128	234	337.6	338.5	0.1112
3	35128	147	216.5	251.2	0.1073
4	35128	190	249.4	262.9	0.0735
5	35128	221	348.0	334.0	0.2307

S3. Figures

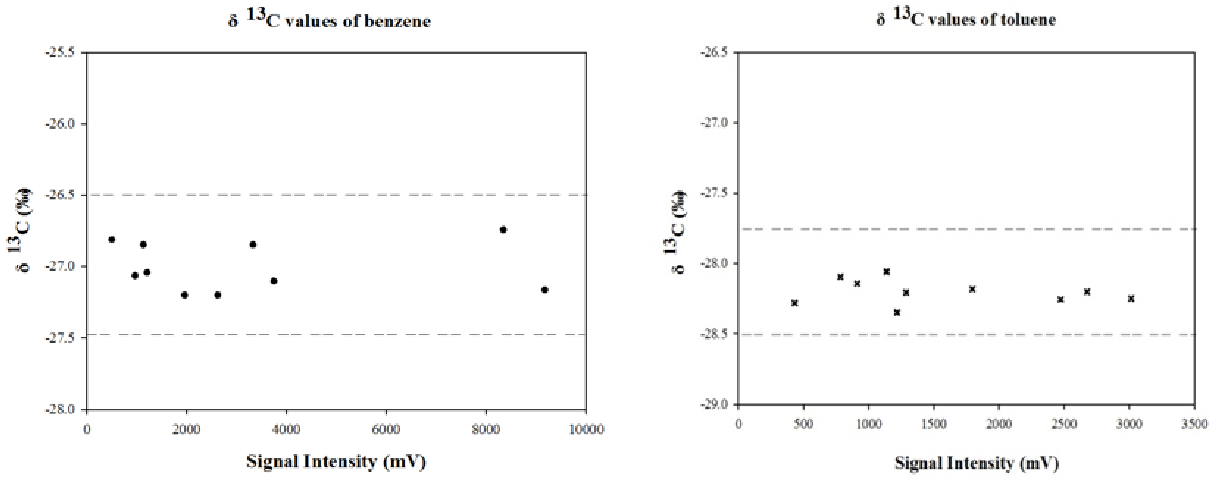

Figure S1. The relationship between δ13C values and signal intensity

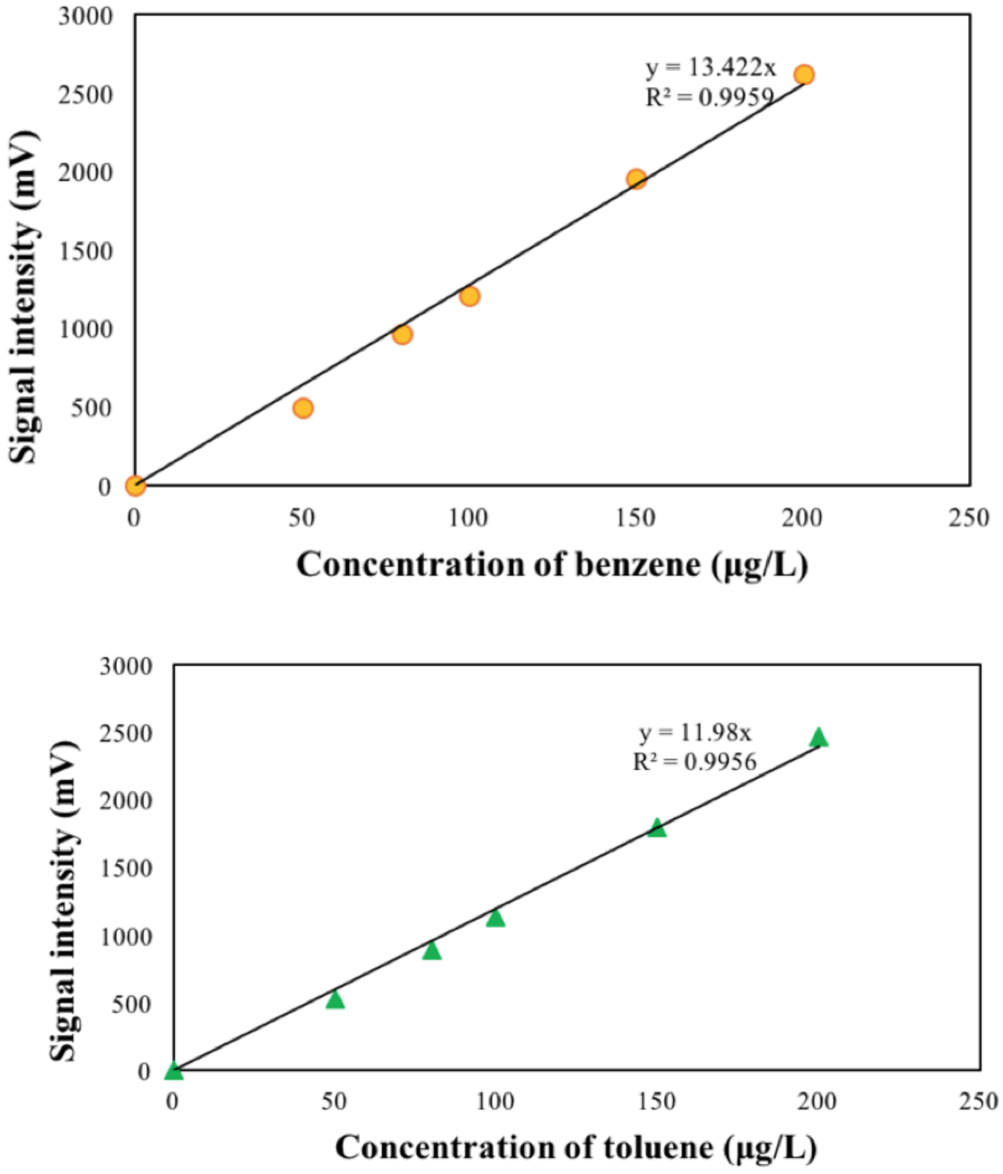

Figure S2. Standard curve of contaminant concentration – signal intensity

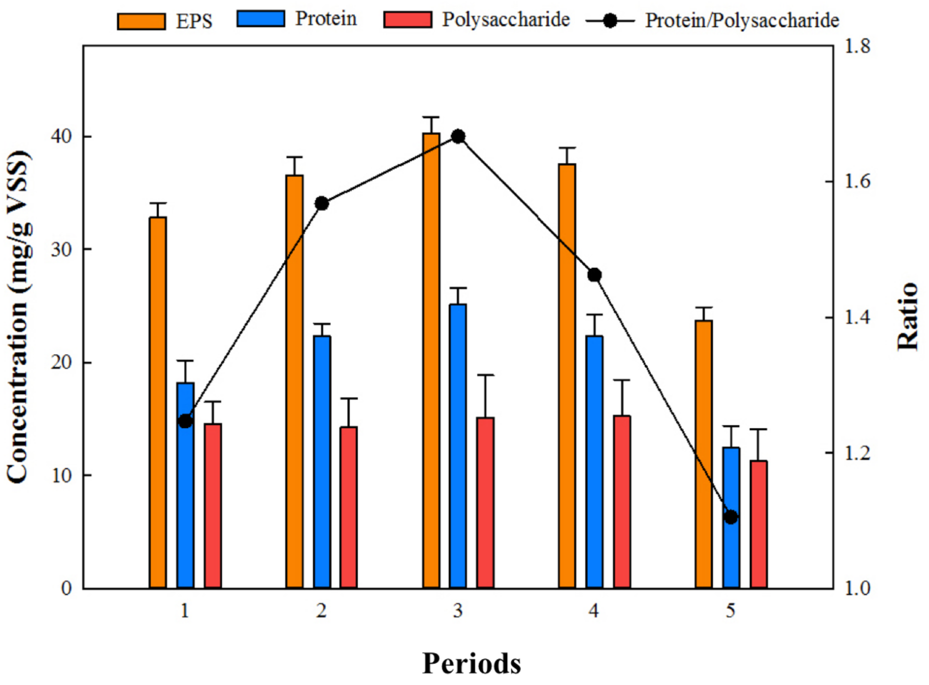

Figure S3. EPS of Bio-PRB in all periods

Extracellular polymeric substances (EPS) are the most important physic-chemical property of biofilms because EPS can indicate the tolerance of microbes to environmental conditions. In Figure S3, the concentrations of EPS were shown for the five experimental periods. EPS showed a rising trend with the increase of influent concentrations, and decreased with the TCE stress.

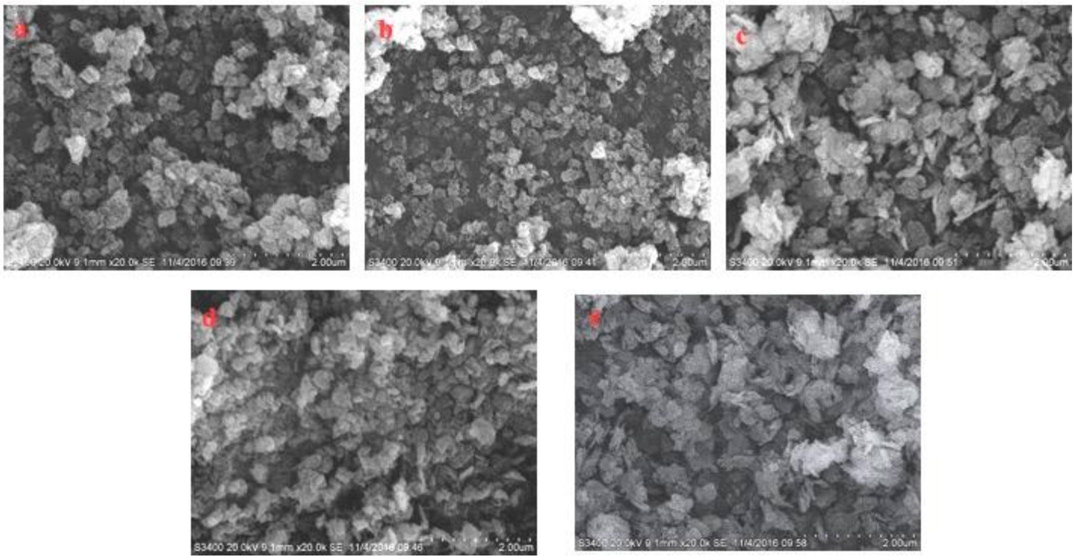

Figure S4. Variation of biofilm in all periods

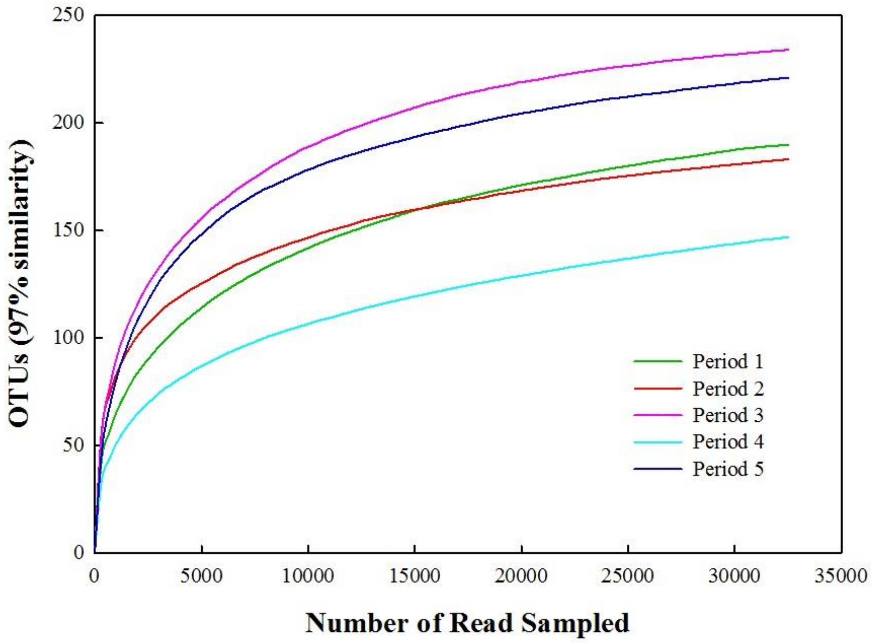

Figure S5. Rarefaction curves of the five activated sludge samples at cutoff level of 3%. The rarefaction curve, plotting the number of observed OTUs as a function of the number of sequences, was computed using RDP's pyrosequencing pipeline.
